# The effect of topically administered lavender aromatherapy on the pain of insulin injection in diabetic patients: a double-blind randomized controlled clinical trial

**DOI:** 10.55730/1300-0144.5531

**Published:** 2022-09-13

**Authors:** Hatice DEMİRAĞ, Sevilay HİNTİSTAN, Enes BULUT

**Affiliations:** 1 Department of Medical Services and Techniques, Kelkit Sema Doğan Vocational School of Health Services, Gümüşhane University, Gümüşhane, Turkey; 2 Department of Nursing, Faculty of Health Sciences, Karadeniz Technical University, Trabzon, Turkey; 3 Department of Emergency Aid and Disaster Management, Faculty of Health Sciences, Artvin Çoruh University, Artvin, Turkey

**Keywords:** Pain, aromatherapy, topical lavender oil, subcutaneous injections

## Abstract

**Background/aim:**

Needle phobia occurs in more than half of diabetic patients due to the pain caused by frequent insulin injections. Therefore, this study evaluated the effect of topically administered lavender aromatherapy on the pain of insulin injections in diabetic patients.

**Materials and methods:**

In this double-blind randomized controlled and experimental study, patients who met the study criteria were divided into three groups; topical lavender oil (n = 60), placebo (n = 60), and control (n = 60) groups. The data were collected using the “Patient Information Form”, the “Follow-up Form”, the “Verbal Category Scale (VCS)”, and the “Visual Analogue Scale (VAS)”.

**Results:**

The results revealed no significant difference between the patients in the topical lavender oil group before and during the insulin injection in terms of VAS and VCS pain scores (p > 0.05). In the placebo and control groups, the mean VAS and VCS pain scores during insulin injection were found to be significantly higher than before insulin injection (p < 0.05). Besides, the mean VAS and VCS scores during insulin injection were significantly higher in the placebo and control groups than the topical lavender oil group (p < 0.05).

**Conclusion:**

The study showed that patients who were administered topical lavender oil felt less pain after insulin injection than those in the placebo and control groups. Therefore, topically applied lavender aromatherapy can be easily used for pain control in insulin-dependent diabetic patients (clinical trial number NCT04767737).

## 1. Introduction

Diabetes Mellitus (DM) is a serious chronic disease that negatively affects the quality of life.^1^ The rapid increase in its prevalence both in our country and around the world has made diabetes a significant health problem in developed and developing countries.^1^,^2^ It is reported that approximately three-quarters (79%) of diabetic patients are reported to live in low and middle-income countries.^3^ According to the International Diabetes Federation’s (IDF) 2019 data, 1 out of every 11 people in the world is diabetic. It is stated that the number of diabetic patients, which is 463 million in the world between the ages of 20–79 in 2019, will reach 700 million by 2045, with an increase of 51%.^4^ It is estimated that this number will reach 16.5 million in 2045 (12.8 million in 2017) in Turkey, and it will become one of the top 10 countries in terms of diabetes.^2^

To control the course of the disease and to reduce its complications, insulin treatment is planned for the whole life of Type 1 diabetic patients, and frequently for approximately 40% of Type 2 diabetic patients [1–4]. Frequent or incorrect administration of insulin injections may lead to the development of complications such as regional pain, ecchymosis, or hematoma on the injection site. It is emphasized that the pain caused by repeated insulin injections negatively affects the psychological well-being of the individuals as well as their physical comfort. Besides, such problems make it challenging to administer repeated injections safely [5–7].

Perception of pain involves various mental processes, such as the individual’s feelings and beliefs about pain [8]. Therefore, early pain experience leads to the development of needle phobia and a poor attitude towards treatment [9]. In a relevant study, 58.5% of diabetic patients were found to have a fear of pain due to insulin injections [10].

Pain control is one of the main tasks of nurses [11]. By alleviating pain, the patient’s acceptance of treatment increases, and thus the quality of life improves [12]. Therefore, nurses should seek different ways of effective pain control. In this sense, complementary therapies are continuously evolving within the health system [13]. The use of complementary therapies reduces the development of complications and the need for synthetic analgesics [14]. In the literature, lavender is reported to have the ability to heal burns and insect bites [15] as well as analgesic [16–18], wound healing [19], antibacterial, antifungal, sedative, and antidepressant effects. The main components of lavender *(Lavandula angustifolia)* essential oil, such as lavender and linalyl acetate, and linalool, are also recommended as topical analgesics in animal models [20].

Whatever the cause, acute pain is a serious problem for individuals with chronic illnesses. Therefore, this study evaluated the effect of topically administered lavender aromatherapy on the pain of insulin injections in diabetic patients.

## 2. Materials and method

### 2.1. Research type

This study is in a double-blind randomized controlled and experimental design and was conducted with diabetic patients receiving insulin therapy.

### 2.2. Setting and time of the study

The study was carried out at the Internal Medicine Service of the TR Ministry of Health X Y State Hospital between May 2020 and July 2020.

### 2.3. The Universe and sample of the research

The population of the research consisted of insulin-dependent diabetic patients over the age of 18 who were hospitalized at the Internal Medicine Service of the TR The Ministry of Health X Y State Hospital between May 2020 and July 2020.

The sample was determined as 159 (53 for each group) patients for three groups (lavender oil group, placebo group, control group) by using the power analysis method (G*Power 3.1.9.6 program), with the margin of error α = 0.05, 0.25 medium effect size, and 0.80 (80%) target strength of the test. However, 7 patients who met the study inclusion criteria were added to each group (60 in the lavender oil group, 60 in the placebo group, 60 in the control group) in case some of the participants quit the study for any reason (Figure). While the patients were randomized, different application groups (topical lavender oil, placebo, control group) were determined by lot in different months (May, June, and July) to minimize the possibility of being affected by each other.

### 2.4. Levander oil

‘Talya’ brand (Talya Herbal Products Tic. San. Ltd. Şti., Antalya, Turkey) 100% pure *L. angustifolia* oil, analyzed in the laboratory of Talya Kimya, was used in the patients. That is, lavender oil was a commercial product supplied from the market.

### 2.5. Inclusion and exclusion criteria of the study

Inclusion criteria for the patients were;

1. being 18 years of age or older,
2. having Type-1 DM or Type-2 DM disease,
3. being conscious and communicating, not having mental and cognitive impairment,
4. not having eczema and fragrance allergy to lavender,
5. not having an alcohol or narcotic addiction,
6. not having a head injury or convulsion history,
7. not having diabetes-related neuropathy, and
8. if the patient took sedatives or analgesics, at least 6 hour would pass,

Exclusion criteria for the patients were;

1. having a history of addiction or diagnosed psychological disorders,
2. having an unstable hemodynamic status,
3. having skin disease symptoms such as wounds and eczema at the insulin injection site and,
4. having an allergy to lavender.

To determine whether the patients in the topical lavender oil group had allergies to lavender oil, 1 drop of 100% lavender oil was dropped on a small area on the inner side of their arms to examine the development of skin reactions such as redness, itching, pain, burning, and sensitivity [21,22]. Patients experiencing these symptoms of reaction were excluded from the study.

### 2.6. Applications for topical lavender oil, placebo, and control groups

Since this study was a double-blind randomized controlled study, no information about the drug (topical lavender oil, topical water) was given to the nurse (researcher) who gave insulin injection, and the patient to reduce bias. The drug to be used was applied in spray bottles without any labels.

Before the administration, pain level, blood pressure, respiratory rate, pulse rate, oxygen saturation level (SPO_2_), and blood glucose of all patients (topical lavender oil, placebo, and control groups) were measured, and then, 3 puffs (0.3 mL) of 100% lavender (*L. angustifolia*) essential oil to the topical lavender oil group and 3 puffs (0.3 mL) of topical distilled water to the placebo group were sprayed on the arms of the patients. No application was applied to the control group. Five minute later, the insulin injection site was wiped with 10% povidone-iodine (baticonol) in all patients, and the injection was given. During the administration of the insülin, the pain levels of the patients were measured again. After giving the injection, blood pressure, respiratory rate, pulse rate, oxygen saturation level, and blood glucose of the patients were also measured again.

To ensure consistency, an insulin injector of 30 gauge and 8 mm needle length (BD Micro-Fine Plus brand) was used, and insulin was applied at a 90° angle by grasping the patient’s arm by hand. During the study, the applications were made to all three groups by the same researcher. One drop of 100% lavender oil was dropped to the researcher’s collar before applying and injecting insulin to all groups so that s/he does not understand what is applied in which group. Besides, the same site (back of the arm) was preferred for all patients for injection.

### 2.7. Data collection forms and administration procedure

To collect the data, the “Patient Information Form” including information about patients’ sociodemographic and medical conditions, the “Topical Lavender Oil/Placebo/Control Group Follow-up Form”, the “Verbal Category Scale (VCS)” and the “Visual Analogue Scale (VAS)” were used.

**The patient information form** was developed by the researchers after reviewing the literature [21,22]. It took approximately 10 min to fill in the form by interviewing each patient before the administration of insulin. The questions in the patient information form were asked to the patients by the researcher, and the answers were recorded in the form. This form includes two parts.

The first part consists of six questions to determine the sociodemographic characteristics of the patients (gender, age, education, marital status, occupation, income perception level).

The second part includes six questions investigating the “medical condition of patients” including diabetes type, duration of diabetes, insulin treatment duration, allergy status, the reason for hospitalization, and the presence of comorbid chronic disease, etc.

**The topical lavender oil/placebo/control group follow-up form** was developed by the researchers to record the pain level (VAS and VCS), blood pressure, respiratory rate, pulse rate, oxygen saturation level, and blood glucose level of the patients before and after the insülin administration.

**Verbal category scale (VCS)** is based on the patient’s choice of the most appropriate word to identify his/her pain [23]. Accordingly, the patient was asked to rate his/her pain between 0–4 as 0; no pain, 1: mild pain, 2: severe pain, 3: very severe pain, 4: unbearable pain.

**Visual analogue scale (VAS)** is used to digitalize the pain level that cannot be measured numerically. A line with a length of 100 mm has the words “no pain” at one end and “the most unbearable pain” on the other. The patient indicates his/her pain by choosing the most appropriate place on the line. Then, the level of pain experienced by the patient is determined by using a scale with scores between 0–10. According to this scoring system, less than 3 points indicate mild pain, 3–6 points indicate moderate pain, and more than 6 points indicate severe pain [24].

### 2.8. Ethical considerations

To conduct the study, required permission was received from Gümüşhane Provincial Health Directorate for Gümüşhane X Hospital (dated 15/06/2020 and numbered 62876282-044-E.1590) and Gümüşhane University Ethics Committee (2020/5 number and 05/05/2020) and was registered with reference number NCT04767737 in the clinical trial management system. Besides, all participants gave informed consent prior to their inclusion in the study after they were informed about the study.

### 2.9. Evaluation of data

Statistical Package for Social Science (SPSS) 24.0 program was used for statistical evaluation of the results. According to the Kolmogorov-Smirnov test based on the number of samples, it was determined that the data were normally distributed. The data were presented as mean, percentage, and standard deviation. Paired sample t-test in the dependent groups, one-way ANOVA test, and post hoc Tukey test in the independent groups and chi-square were used to analyze the data. p < 0.05 was considered as statistically significant.

### 2.10. Conflict of interest and financing

In this study, there is no conflict of interest between the authors or with any company. This study was not supported by any research fund.

## 3. Results

This study was conducted with 180 patients who met the research criteria. More than half of the participants in the study were male (52.2%), the majority of them were married (71.1%), they had primary school education and below (66.7%), 38.3% of them were farmers, and most of them had Type 2 diabetes (81.1%). Almost no participants had any allergies (90.6%), and half had comorbid chronic disease other than diabetes (50.0%) (Table 1).

The chi-square test, which was used to compare the topical lavender oil, placebo, and control group patients according to their sociodemographic characteristics, revealed no statistically significant difference in terms of gender, marital status, education level, diabetes type, allergy status, and having a comorbid chronic disease (p > 0.05). A statistically significant difference was found between the distribution of patients in the topical lavender oil, placebo, and control groups in terms of their occupations (X^2^ = 10.633; p = 0.031) (p > 0.05) (Table 1).

The distribution of the patients in the topical lavender oil, placebo, and control groups according to their medical conditions and vital signs is presented in Table 2. The mean age of the participants in the study was 52.82 ± 13.48 years, the mean blood glucose level was 172.74 ± 44.54 mg/dL, the mean duration of diabetes was 14.37 ± 12.22 years, and the mean duration of insulin use was 9.13 ± 12.24 years. The mean measurements of the patients before topical lavender oil application and insulin injection were as follows; systolic blood pressure 117.63 ± 19.50 mm/Hg, diastolic blood pressure 71.16 ± 10.47 mm/Hg, pulse rate 86.86 ± 17.29 min, respiration 21.67 ± 2.52 min, SPO_2_ 97.47 ± 5.72 (%), the VCS was 0.46 ± 0.67, and the VAS was 0.88 ± 1.09. After the insulin injection, the mean systolic blood pressure was found to be 116.27 ± 13.75 mm/Hg, the mean diastolic blood pressure was 71.97 ± 8.72 mm/Hg, the mean pulse rate was 87.81 ± 13.77 min, the mean respiratory rate was 21.49 ± 2.00 min, and the mean SPO_2_ was 97.97 ± 1.37 (%). During the insulin injection, the mean VCS was determined to be 1.09 ± 0.78, and the mean VAS was 2.16 ± 1.72 (Table 2).

The results of the one-way ANOVA and post hoc analysis tests for the comparison of topical lavender oil, placebo, and control groups according to the medical condition and vital signs of the patients showed that the mean diastolic blood pressure of the patients in the control group (75.83 ± 8.29) after insulin injection was significantly higher than topical lavender oil (70.50 ± 8.71) and placebo (69.58 ± 7.93) group (F = 9.869; p = 0.000) (p < 0.05). Post hoc analysis showed that this significance between them was due to the control group. It was also determined that the mean respiration after insulin injection was significantly higher in the control group (22.41 ± 1.70) than the topical lavender oil (21.06 ± 2.23) and placebo (21.00 ± 1.73) groups (F = 10.532; p = 0.000) (p < 0.05). Post hoc analysis showed that this significance between them was due to the control group and placebo group. Besides, after insulin injection, the mean SPO_2_ was statistically significantly lower in the control group (97.36 ± 1.30) compared to the topical lavender oil (98.55 ± 1.32) and placebo (98.00 ± 1.24) groups (F = 12.633; p = 0.000) (p < .05). Post hoc analysis showed that this significance between them was due to the control group.

The mean VCS score, which shows the severity of pain during insulin injection, was significantly lower in the topical lavender oil group (0.55 ± 0.64) compared to the placebo (1.25 ± 0.68) and control (1.48 ± 0.72) groups (F = 30.157; p = 0.000) (p < 0.05). A significant difference was also seen in the mean VAS scores, which shows the severity of pain during insulin administration, among the topical lavender oil group (1.10 ± 1.28), the placebo group (2.27 ± 1.53), and the control group (3.12 ± 1.70) (F = 26.597; p = 0.000) (p < 0.05). The post hoc analysis showed that this difference was caused by the placebo and control groups (Table 2).

The one-way ANOVA test performed for the comparison of the medical condition and vital signs of the patients in the topical lavender oil, placebo, and control groups revealed no significant difference among these groups in terms of age, blood glucose, duration of diabetes, duration of insulin use, systolic blood pressure before insulin injection, diastolic blood pressure, pulse, respiration, SPO_2_, VCS score, VAS score, systolic blood pressure after insulin injection and the mean pulse rate after the application (p > 0.05) (Table 2).

Paired sample t-test, which was performed to compare the mean scores and VCS and VAS before and during insulin injection of topical lavender oil, placebo, and control groups, demonstrated that the mean VCS pain score of the placebo (t = −12.907; p = 0.000), and control (t = −3.563; p = 0.000) groups increased significantly during the administration compared to VCS score before the administration (p < 0.05). The mean VAS pain scores of the placebo (t = −9.530; p = 0.000) and control (t = −12.780; p = 0.000) groups during the administration were also significantly higher than the mean VAS pain scores before the administration (p < 0.05). However, no significant difference was found between the mean VCS and VAS pain scores of the patients in the topical lavender oil group before and during insulin injection (p > 0.05) (Table 3).

## 4. Discussion

This double-blind randomized controlled study investigated the effect of topically administrated lavender aromatherapy on the pain of insulin injection in diabetic patients.

Frequently used in aromatherapy, *L. angustifolia*, one of the complementary treatments, contains many active ingredients. “Linalool” and “linalyl acetate” are among these active ingredients, and linalool acts as a sedative by affecting gamma-aminobutyric acid receptors. Another active ingredient, linalyl acetate, has a narcotic function [25]. There are various studies in the literature examining the effect of aromatherapy on pain caused by the insertion of an intravenous catheter, injection insertion into the forearm, or arteriovenous (AV) fistula application via injection [16,21,22,26–29]. However, no national or international studies to our knowledge have investigated the use of topical aromatherapy during insulin injection in diabetic patients. The vital signs of the patients in the study were measured as follows before insulin injection; systolic blood pressure 117.63 ± 19.50 mm/Hg, diastolic blood pressure 71.16 ± 10.47 mm/Hg, pulse rate 86.86 ± 17.29 min, respiration 21.67 ± 2.52 min, and SPO_2_ 97.47 ± 5.72 (%). Measurements after insulin injection were found to be as follows; the mean systolic blood pressure 116.27 ± 13.75 mm/Hg, the mean diastolic blood pressure 71.97 ± 8.72 mm/Hg, the mean pulse rate 87.81 ± 13.77 min., the mean respiration 21.49 ± 2.00 min, and the mean SPO_2_ 97.97 ± 1.37(%). Besides, a statistically significant difference was seen between the groups in terms of diastolic blood pressure, respiration, and SPO_2_. Tüzün Özdemir [22] examined the effect of aromatherapy on the pain of injection of an AV fistula and found the mean systolic blood pressure of the patients participating in the study as 139.99 mm/Hg, the mean diastolic blood pressure as 81.24 mm/Hg, and the mean pulse rate as 81.39 min. Contrary to our study, there was no statistically significant difference between the groups. That the mean systolic blood pressure and diastolic blood pressure scores in our study were found to be lower than those of Tüzün Özdemir’s [22] study is thought to be due to the effect of aromatherapy on hypertension associated with cardiovascular disease [30–32]. In parallel with the literature reviewed, patients in the control group in this study had significantly higher mean diastolic blood pressure after insulin injection [22].

In our study, no significant difference was found between the VCS and VAS pain scores of patients in the topical lavender oil group before and during insulin injection. However, it was found that the mean VCS and VAS pain scores of the patients in the placebo and control groups during insulin injection were significantly higher than before insulin injection. In addition, mean VCS and VAS scores during insulin injection were significantly higher in the placebo and control groups than in the topical lavender oil group. Although there was no significant difference in the mean pain scores before and during the application of insulin in the topical lavender oil group (VCS t = −0.228, p = 0.821; VAS t = −0.314, p = 0.755), we can say that topically applied aromatherapy was effective in reducing pain due to the statistically increased pain in the placebo and control groups and the significant difference in pain scores during the application of insulin between groups. It is emphasized in the relevant literature that both inhaler [22,26,33] and topical lavender oil aromatherapy [34] significantly reduce pain during AV fistula puncture. In some other studies, lavender aromatherapy was found to significantly reduce the total pain score at the 5th and 10th min of intravenous catheter insertion in both children [28] and adults [29]. In a study examining the effect of lavender aromatherapy on the pain felt during the injection, it was reported that inhaled lavender aromatherapy applied significantly reduced pain [16]. In an experimental study, Aliasgharpour et al. [21] measured VAS pain scores by applying 3 consecutive sessions of lavender aromatherapy in hemodialysis patients and reported that the pain during puncture of the arteriovenous fistula was 5.36 ± 2.08, 5.69 ± 2.29, and 5.58 ± 2.15 in the control group, respectively and 4.00 ± 2.48, 3.05 ± 1.94, 2.97 ± 2.27 in the experimental group, respectively [21]. In another study examining the effect of lavender aromatherapy on pain during puncture of arteriovenous fistula, it was reported that the mean VAS pain severity score in the experimental groups was 3.78 ± 0.24 before the application and decreased significantly to 2.36 ± 0.25 after the application [27]. In our study, the mean pain score of the topical lavender oil group was found to be lower than the other studies reviewed. The reason for this is estimated to be the size of the injection used during the insulin administration (insulin injector with 30 gauge and 8 mm length) and the frequency of insulin application of the patients.

### 4.1. Limitations

In this study, the short-term effect of lavender essential oil has been evaluated. Therefore, the findings obtained are valid for the short-term effect of lavender essential oil on pain. The results of this study cannot be generalized because 1 Internal Medicine Service was involved in the study. Moreover, another limitation is that a placebo control group was not used in this study.

## 5. Conclusion

The findings of this study showed that patients who were applied topical lavender oil during insulin injection felt less pain than the placebo and control group patients. As a result, it is concluded that topical lavender aromatherapy can be easily applied for pain control in insulin-dependent diabetic patients. Nurses or healthcare professionals are recommended to regularly assess patients’ pain caused by insulin administration and include aromatherapy in their nursing practices.

## Figures and Tables

**Figure:**
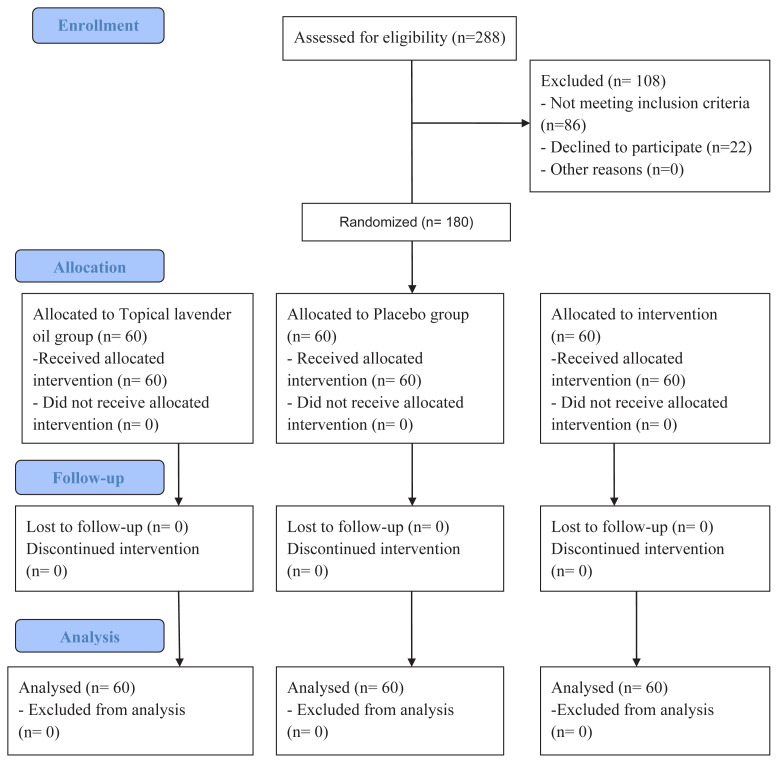
The study flow diagram.

**Table 1:** Distribution of the sociodemographic and disease-related characteristics of diabetic patients (n = 180).

Variable		N (%)				Chi-square test	
		Topical lavender oil	Plasebo	Control	Total	X^2^	P
**Gender**	Male	34(56.7)	29(48.3)	31(51.7)	94(52.2)	0.846	0.655
	Female	26(43.3)	31(51.7)	29(48.3)	86(47.8)		
**Marital status**	Married	45(75.0)	43(71.7)	40(66.7)	128(71.1)	1.028	0.598
	Single (divorced/widow(er))	15(25.0)	17(28.3)	20(33.3)	52(28.9)		
**Education level**	Primary+secondary school and below	45(75.0)	38(63.3)	37(61.7)	120(66.7)	6.031	0.197
	High school	10(16.7)	11(18.3)	17(28.3)	38(21.1)		
	University and above	5(8.3)	11(18.3)	6(10.0)	22(12.2)		
**Occupation**	Housewife/self-employed/retired	12(20.0)	27(45.0)	18(30.0)	57(31.7)	**10**.**633**	**0**.031*
	Farmer	29(46.3)	15(25.0)	25(41.7)	69(38.3)		
	Worker-civil servant	19(31.7)	18(30.0)	17(28.3)	54(30.0)		
**Type of diabetes**	Type 1 diabetes	10(16.7)	13(21.7)	11(18.3)	34(18.9)	0.508	0.776
	Type 2 diabetes	50(83.3)	47(78.3)	49(81.7)	146(81.1)		
**The presence of allergy**	Yes	5(8.3)	7(11.7)	5(8.3)	17(9.4)	0.520	0.771
	No	55(91.7)	53(88.3)	55(91.7)	163(90.6)		
**The presence of comorbid disease**	Yes	29(48.3)	30(50.0)	31(51.7)	90(50.0)	0.133	0.936
	No	31(51.7)	30(50.0)	29(48.3)	90(50.0)		

* p < 0.05

**Table 2:** Distribution of medical status and vital signs results of diabetic patients (n = 180).

Variable	Mean ± SD				One-way ANOVA	
	Topical lavender oil	Placebo	Control	Total	F	P
Age (year)	50.91 ± 13.42	53.80 ± 13.65	53.76 ± 13.41	52.82 ± 13.48	0.903	0.407
Blood glucose (mg/dL)	170.33 ± 49.98	178.45 ± 45.58	169.45 ± 37.31	172.74 ± 44.54	0.742	0.478
Duration of diabetes (year )	14.32 ± 12.93	15.15 ± 12.44	13.65 ± 11.39	14.37 ± 12.22	0.225	0.799
Duration of insulin use (year)	7.87 ± 12.30	10.18 ± 12.77	9.35 ± 11.73	9.13 ± 12.24	0.548	0.579
Systolic BP (mm/Hg) before insulin injection	116.83 ± 20.54	119.41 ± 18.59	116.66 ± 19.54	117.63 ± 19.50	0.372	0.690
Systolic BP (mm/Hg) after insulin injection	116.33 ± 15.29	113.50 ± 11.50	119.00 ± 13.86	116.27 ± 13.75	2.438	0.090
Diastolic BP (mm/Hg) before insulin injection	70.50 ± 9.81	71.16 ± 11.65	71.83 ± 9.99	71.16 ± 10.47	0.241	0.786
Diastolic BP (mm/Hg) after insulin injection	70.50 ± 8.71	69.58 ± 7.93	75.83 ± 8.29	71.97 ± 8.72	**9**.**869**	**0**.000*
Pulse before insulin injection (min)	89.20 ± 18.09	91.41 ± 17.78	88.96 ± 16.10	86.86 ± 17.29	0.364	0.695
Pulse after insulin injection (min)	85.11 ± 14.56	87.51 ± 13.50	90.80 ± 12.82	87.81 ± 13.77	2.620	0.076
Respiration before insulin injection (min)	22.00 ± 2.55	21.56 ± 2.56	21.46 ± 2.45	21.67 ± 2.52	0.757	0.471
Respiration before insulin injection (min)	21.06 ± 2.23	21.00 ± 1.73	22.41 ± 1.70	21.49 ± 2.00	**10**.**532**	**0**.000**
SPO_2_ before insulin injection (%)	96.60 ± 9.65	97.81 ± 1.73	98.00 ± 1.51	97.47 ± 5.72	1.059	0.349
SPO_2_ after insulin injection (%)	98.55 ± 1.32	98.00 ± 1.24	97.36 ± 1.30	97.97 ± 1.37	**12**.**633**	**0**.000**
VCS score before insulin injection	0.53 ± 0.72	0.45 ± 0.67	0.40 ± 0.61	0.46 ± 0.67	0.601	0.549
VCS score during insulin injection	0.55 ± 0.64	1.25 ± 0.68	1.48 ± 0.72	1.09 ± 0.78	**30**.**157**	**0**.000**
VAS score before insulin injection	1.07 ± 1.16	0.87 ± 1.09	0.70 ± 1.01	0.88 ± 1.09	1.694	0.187
VAS score during insulin injection	1.10 ± 1.28	2.27 ± 1.53	3.12 ± 1.70	2.16 ± 1.72	**26**.**597**	**0**.000*

BP: Blood pressure, SPO_2_: Oxygen saturation, VCS: Verbal category scale, VAS: Visual analogue scale

* p < 0.001,

** p < 0.001 and post hoc Tukey test,

**Note:** In repeated measurements n = 180

**Table 3:** Distribution of diabetes patients’ VCS and VAS mean scores (n = 180).

Variable	Mean ±SD		
	Topical lavender oil	Placebo	Control
VCS score before insulin injection	0.53 ± 0.72	0.45 ± 0.67	0.40 ± 0.61
VCS score during insulin injection	0.55 ± 0.64	1.25 ± 0.68	1.48 ± 0.72
**Paired sample t-test**	t = −0.228 P = 0.821	**t =** −**12**.**907** **P = 0**.000*	**t =** −**13**.**563** **P = 0**.000*
VAS score before insulin injection	1.07 ± 1.16	0.87 ± 1.09	0.70 ± 1.01
VAS score during insulin injection	1.10 ± 1.28	2.27 ± 1.53	3.12 ± 1.70
**Paired sample t-test**	t = −0.314	**t =** −**9**.**530**	**t =** −**12**.780*
	P = 0.755	**P = 0**.000*	**P = 0**.000*

* p < 0.001

## Footnotes

1 Türkiye Endokrinoloji ve Metabolizma Derneği (2019). Diabetes Mellitus Çalışma ve Eğitim Grubu. Diabetes Mellitus ve Komplikasyonlarının Tanı, Tedavi ve İzlem Kılavuzu [online]. Website http://temd.org.tr/admin/uploads/tbl_kilavuz/20190819095854-2019tbl_kilavuzb48da47363.pdf. [accested 25 March 2019] (in Turkish).

2 International Diabetes Federation (2018). Diabetes atlas [online]. Website http://www.diabetesatlas.org/key-messages.html. [accested 25 March 2019].

3 International Diabetes Federation (2018). Diabetes. Europe: Prevalence of diabetes (20–79 years), 2019 [online]. Website https://www.diabetesatlas.org/upload/resources/material/20191218_144548_eur_factsheet_en.pdf. [accested 25 March 2019].

4 International Diabetes Federation (2019). Diabetes Atlas. GLOBAL Fact sheet: Number of adults (20–79 years) with diabetes worldwide [online]. Website https://www.diabetesatlas.org/upload/resources/material/20191218_144459_2019_global_factsheet.pdf. [accested 25 March 2019].
